# Effects of Subchronic Buspirone Treatment on Depressive Profile in Socially Isolated Rats: Implication of Early Life Experience on 5-HT1A Receptor-Related Depression

**DOI:** 10.3390/ph17060717

**Published:** 2024-06-01

**Authors:** Nian-Sheng Tzeng, Jing-Yi Chung, Chen-Cheng Lin, Pao-Yun Cheng, Yia-Ping Liu

**Affiliations:** 1 Department of Psychiatry, Tri-Service General Hospital, Taipei 114, Taiwan; pierrens@mail.ndmctsgh.edu.tw; 2 Student Counseling Center, National Defense Medical Center, Taipei 114, Taiwan; 3 Laboratory of Cognitive Neuroscience, Department of Physiology and Biophysics, National Defense Medical Center, Taipei 114, Taiwan; minnie-house@hotmail.com (J.-Y.C.); wsadhjkl@gmail.com (C.-C.L.); pycheng@mail.ndmctsgh.edu.tw (P.-Y.C.); 4 Department of Psychiatry, Cheng Hsin General Hospital, Taipei 112, Taiwan

**Keywords:** 5-HT1A receptors, 8-OH-DAPT, buspirone, depression, development, isolation rearing, partial agonism

## Abstract

The heterogeneity of etiology may serve as a crucial factor in the challenges of treatment, including the low response rate and the delay in establishing therapeutic effect. In the present study, we examined whether social experience since early life is one of the etiologies, with the involvement of the 5-HT1A receptors, and explored the potentially therapeutic action of the subchronic administration of buspirone, a partial 5-HT1A agonist. Rats were isolation reared (IR) since their weaning, and the depressive profile indexed by the forced-swim test (FST) was examined in adulthood. Nonspecific locomotor activity was used for the IR validation. Buspirone administration (1 mg/kg/day) was introduced for 14 days (week 9–11). The immobility score of the FST was examined before and after the buspirone administration. Tissue levels of serotonin (5-HT) and its metabolite 5-HIAA were measured in the hippocampus, the amygdala, and the prefrontal cortex. Efflux levels of 5-HT, dopamine (DA), and norepinephrine (NE) were detected in the hippocampus by brain dialysis. Finally, the full 5-HT1A agonist 8-OH-DPAT (0.5 mg/kg) was acutely administered in both behavioral testing and the dialysis experiment. Our results showed (i) increased immobility time in the FST for the IR rats as compared to the social controls, which could not be reversed by the buspirone administration; (ii) IR-induced FST immobility in rats receiving buspirone was corrected by the 8-OH-DPAT; and (iii) IR-induced reduction in hippocampal 5-HT levels can be reversed by the buspirone administration. Our data indicated the 5-HT1A receptor-linked early life social experience as one of the mechanisms of later life depressive mood.

## 1. Introduction

Sinking into a depressive mood is a common psychological state that occurs in many people during their lives. It can be long-term torture for a person or family. Currently, the treatment of depressive disorders is diversified, covering pharmacological and non-pharmacological paradigms. However, the overall outcome is not as expected. For pharmacological intervention, about 40–50% of the patients that initially received antidepressant treatment are unable to reach timely remission [1], and up to one-fourth of the patients still fail to respond even after two or more antidepressants are prescribed, which is defined as treatment-resistant depression (TRD), and that causes a huge socioeconomic burden [2]. The underlying reason is complicated, possibly highly relevant to its multiple etiological origins (i.e., the heterogeneous nature of depressive disorders), including what happens during development. 

For investigating the developmental etiology of mental disorders, the preclinical animal method is useful so as to provide a more detailed mechanism [3], in which social manipulations with less consideration of developmental origins were found sometimes ineffectively to present a depressive profile. For example, learned helplessness is unable to be induced by social instability intervention (assessed by the forced-swim test, FST, see [4]) and short-term adulthood social deprivation (assessed by the active avoidance test, see [5]). In contrast, ongoing social isolation since early life (i.e., isolation rearing, IR) has been widely employed to examine how the early life social experience affects the individual mood in their adulthood by its value in consistently presenting depression-like behavior by exhibiting a higher immobility score in the FST [6]. There are two key issues worth being highlighted in IR-induced adulthood depression: (i) Isolation time must be long enough in an ongoing manner, and (ii) it has to start within the critical period, i.e., immediately after weaning [7]. Interestingly, both issues require the involvement of monoaminergic neural substrates, including serotonin [8].

The central serotonin (5-HT) system has been implicated in neural regulation with significant developmental and anatomical relevance [9,10,11,12]. For development, the 5-HT1A receptor is involved in neurite branching during the critical period [13] and in the regulation of the IR-induced behavioral phenotypes [14,15,16]. The roles of the 5-HT1A receptors are also anatomically area-dependent, via their presynaptic somatodendritic autoreceptors on a 5-HT cell body and their postsynaptic receptors on projective terminal areas [17,18]. 

Buspirone, like other partial 5-HT1A agonists (for example, gepirone and ipsapirone), appears less effective to treat human anxiety/depression as it did in animal paradigms [18]. The inadequate translational value indicates that the high heterogeneity of clinical mood problems is far beyond the mechanism of the 5-HT1A receptors. On the other hand, it also implies that the manipulation of the partial 5-HT1A receptors may be pharmacologically justified in a more specific milieu. For example, buspirone helps treat depressive moods by accelerating the effects of the SSRIs [19,20]. In this regard, a preclinical buspirone investigation would be beneficial if targeting the 5-HT1A mechanism in the mood-associated brain areas by approaching the dual pharmacological properties of the drug, i.e., partial agonism at the 5-HT1A post-synaptic receptors and full agonism at the 5-HT1A pre-synaptic receptors [19]. So far, there is inadequate knowledge of the 5-HT1A functions regarding the buspirone effects on the rodent social isolation paradigms. Tung and colleagues demonstrated that the IR may modify the 5-HT1A receptor-associated ability to control impulsiveness [14], whereas Frances and Lienard reported buspirone failed to correct the deficit of social behavior due to the isolation housing (i.e., non-developmental origin)-induced hyper-reactivity [20]. Until now, there has been no evidence directly targeting the buspirone effects on the depressive profile in IR rats. 

Aiming to fulfill this knowledge gap, the present study employed IR rats to receive a 14 days buspirone administration. The effects of social deprivation since early life (via IR) and the manipulation of 5-HT1A receptors (via buspirone) were assessed in two domains. Behaviorally, we examined the rats’ performance of the FST to index the helplessness component of depressive behavior, and neurochemically, we examined the tissue concentrations of 5-HT and its metabolite 5-HIAA in three areas highly related to depression, i.e., the hippocampus, prefrontal cortex, and amygdala, so as to explore the possible involvement of the postsynaptic 5-HT1A receptors. The 14 day regimen was chosen because it provides better validity to model the adequate onset time for antidepressants to work for treating depressive moods. Acute 8-OH-DPAT administration was also employed in the present study to examine the 5-HT1A effects on the rats’ FST performance; 8-OH-DPAT exerts 5-HT1A full agonism on the postsynaptic side, in contrast to the partial agonism of buspirone. The results obtained from the present study may shed some new light on the involvement of early life social experience in the 5-HT1A receptor-related psychopathology of depression.

## 2. Results

**Figure 1:**
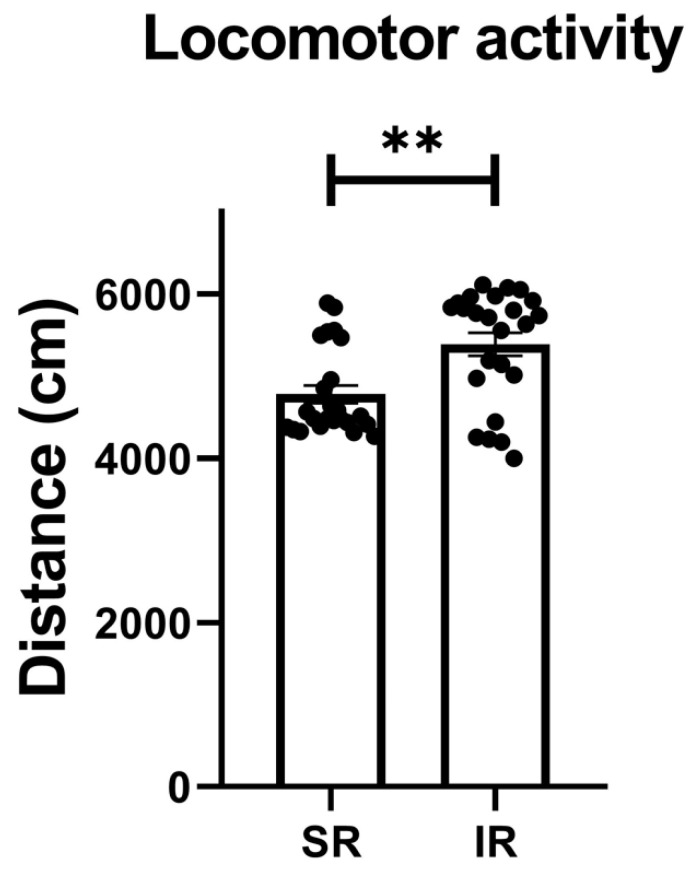
The locomotor activity significantly increased after the 5-week (postnatal weeks 3–8) IR procedure. The data are presented as the mean ± SEM. *n* = 24 for each group. ** *p* < 0.01.

**Figure 2:**
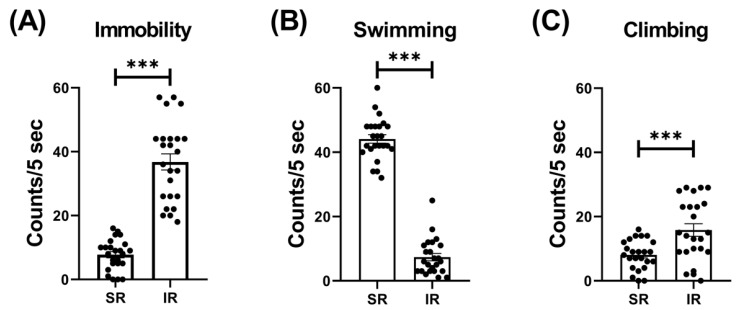
The 5-week (postnatal weeks 3–8) IR procedure significantly increased the immobility score while decreasing swimming and climbing scores. The data are presented as the mean ± SEM. *n* = 24 for each group. *** *p* < 0.001.

The IR group exhibited increased locomotor activity (t_(46)_ = 3.426, *p* = 0.0013) (Figure 1). In terms of the IR effect on the FST performances, the IR group exhibited increased immobility (t_(46)_ = 10.68, *p* < 0.001) (Figure 2A), decreased swimming (t_(46)_ = 20.95, *p* < 0.001) (Figure 2B), and increased climbing (t_(46)_ = 3.597, *p* < 0.001) when compared to the SR group (Figure 2C).

**Figure 3:**
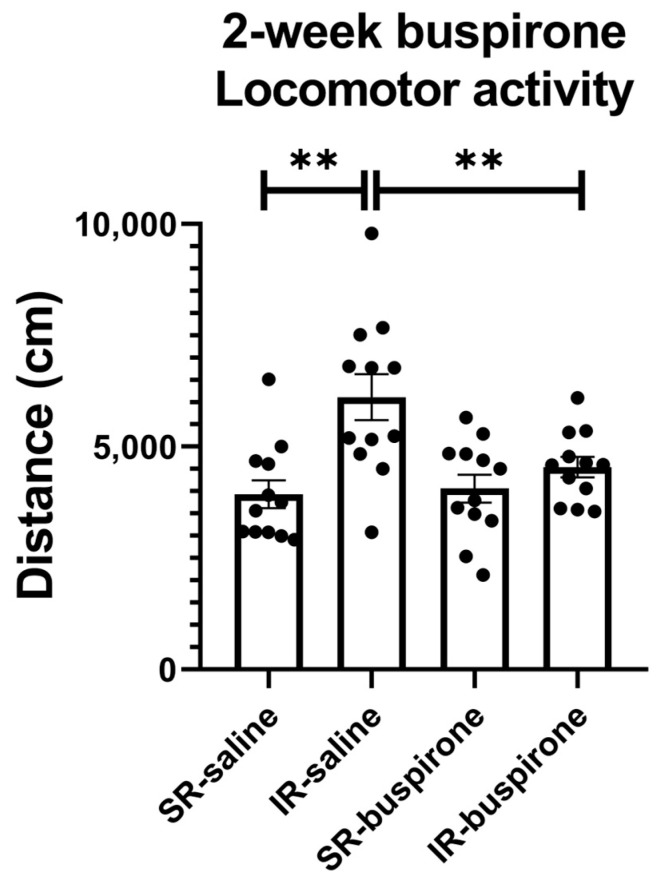
The 2-week buspirone treatment reversed IR-increased locomotor activity. The data are presented as the mean ± SEM. *n* = 12 for each group. ** *p* < 0.01.

For the locomotor activity after the 2-week buspirone treatment, the two-way ANOVA revealed a significant interaction between the “IR” and “buspirone” (F_(1, 44)_ = 5.573, *p* = 0.023). This interaction was driven by the differences observed between the SR-saline and IR-saline groups (F_(1, 44)_ = 18.365, *p* < 0.01), and the SR-buspirone and IR-buspirone groups (F_(1, 44)_ = 9.541, *p* < 0.01) (Figure 3).

**Figure 4:**
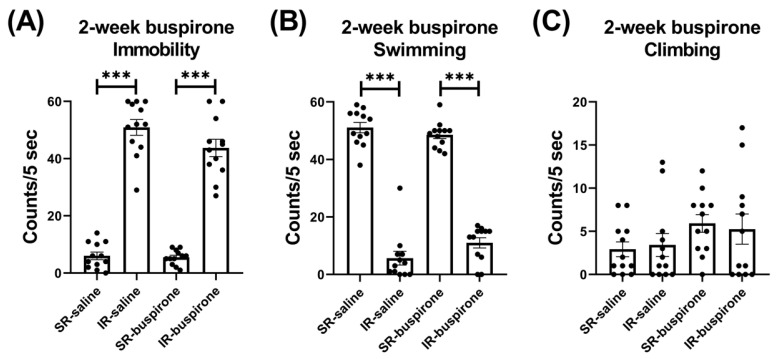
The 2-week buspirone treatment reversed the increase in immobility score and the decrease in swimming score induced by IR. The data are presented as the mean ± SEM. n = 12 for each group. *** *p* < 0.001.

For the immobility score after the 2-week buspirone treatment, the two-way ANOVA revealed a significant main effect on the “IR” (F_(1, 44)_ = 361.992, *p* < 0.001). This main effect was driven by the differences observed between the SR-saline and IR-saline groups (F_(1, 22)_ = 217.057, *p* < 0.001), and the SR-buspirone and IR-buspirone groups (F_(1, 22)_ = 149.102, *p* < 0.001) (Figure 4A). For the swimming score after the 2-week buspirone treatment, the two-way ANOVA revealed a significant interaction between the “IR” and “buspirone” (F_(1, 44)_ = 4.386, *p* = 0.042). This interaction was driven by the differences observed between the SR-saline and IR-saline groups (F_(1, 44)_ = 294.905, *p* < 0.001), and the SR-buspirone and IR-buspirone groups (F_(1, 44)_ = 201.95, *p* < 0.001) (Figure 4B). For the climbing score after the 2-week buspirone treatment, the two-way ANOVA revealed no significant interaction or main effect between the “IR” and “buspirone” (Figure 4C).

**Figure 5:**
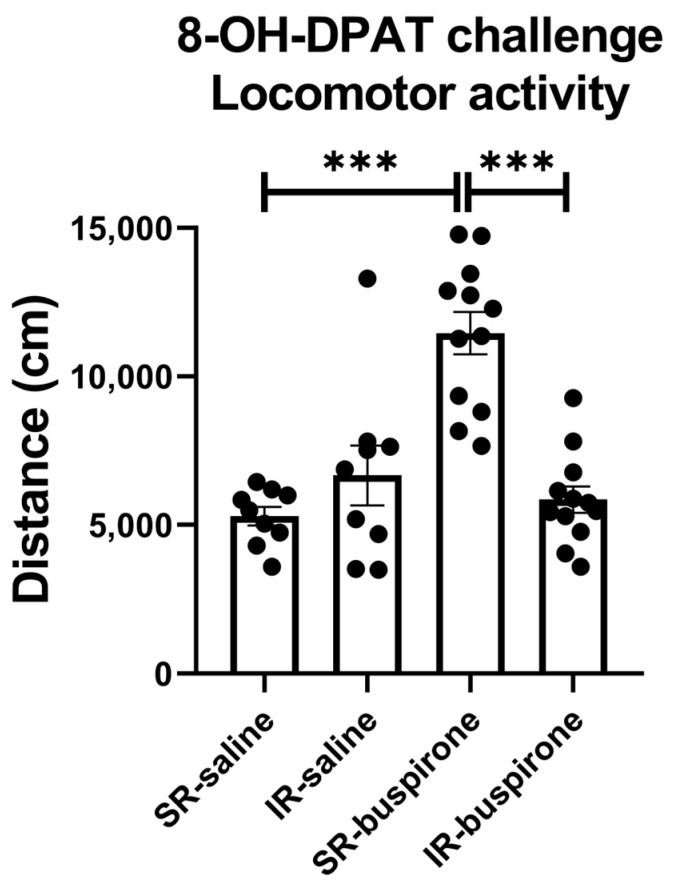
After the acute 8-OH-DPAT challenge, locomotor activity was significantly increased in the SR-saline group. Two-week buspirone treatment reversed IR-increased locomotor activity. The data are presented as the mean ± SEM. *n* = 9 for SR-saline and IR-saline, *n* = 12 for SR-buspirone and IR-buspirone, *** *p* < 0.001.

For the locomotor activity after the acute 8-OH-DAPT challenge, the two-way ANOVA revealed a significant interaction between the “IR” and “buspirone” (F_(1, 38)_ = 27.405, *p* < 0.001). This interaction was driven by the differences observed between the SR-saline and SR-buspirone groups (F_(1, 38)_ = 42.732, *p* < 0.001), and the SR-buspirone and IR-buspirone groups (F_(1, 38)_ = 41.243, *p* < 0.001) (Figure 5).

**Figure 6:**
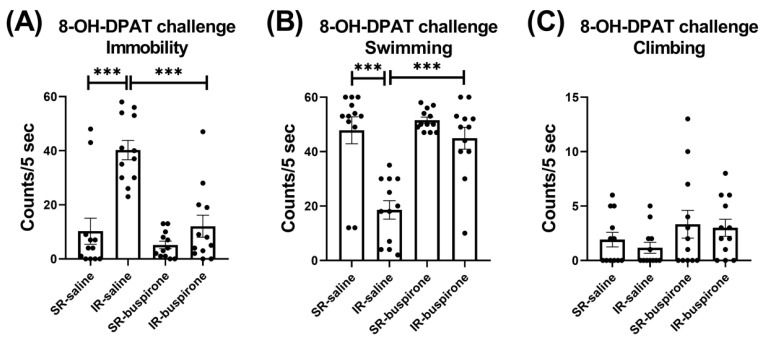
The improvement effects of buspirone on the increased immobility behavior and decreased swimming behavior induced by IR were counteracted by the acute 8-OH-DPAT challenge. The data are presented as the mean ± SEM. *n* = 12 for each group. *** *p* < 0.001.

For the immobility score after the acute 8-OH-DAPT challenge, the two-way ANOVA revealed a significant interaction between the “IR” and “buspirone” (F_(1, 44)_ = 9.727, *p* = 0.003). This interaction was driven by the differences observed between the SR-saline and IR-saline groups (F_(1, 44)_ = 32.858, *p* < 0.001), and the IR-saline and IR-buspirone groups (F_(1, 44)_ = 28.965, *p* < 0.001) (Figure 6A). For the swimming score after the acute 8-OH-DAPT challenge, the two-way ANOVA revealed a significant interaction between the “IR” and “buspirone” (F_(1, 44)_ = 9.573, *p* = 0.003). This interaction was driven by the differences observed between the SR-saline and IR-saline groups (F_(1, 44)_ = 31.882, *p* < 0.001), and the SR-buspirone and IR-buspirone groups (F_(1, 44)_ = 25.841, *p* < 0.001) (Figure 6B). For the climbing score after the acute 8-OH-DAPT challenge, the two-way ANOVA revealed no significant interaction or main effect between the “IR” and “buspirone” (Figure 6C).

**Figure 7:**
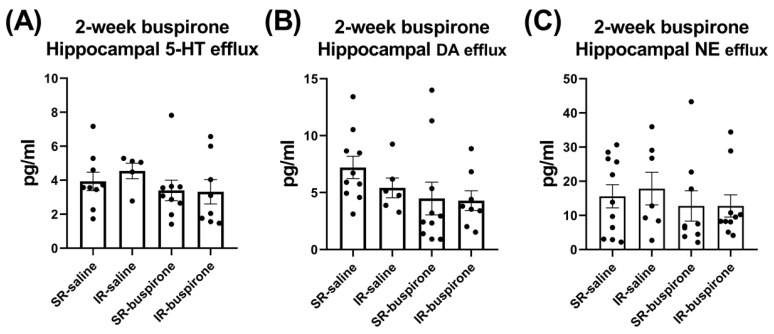
IR and buspirone showed no effect on the efflux of 5-HT, DA, and NE in the hippocampus. The data are presented as the mean ± SEM. *n* = 5–10 for each group.

**Figure 8:**
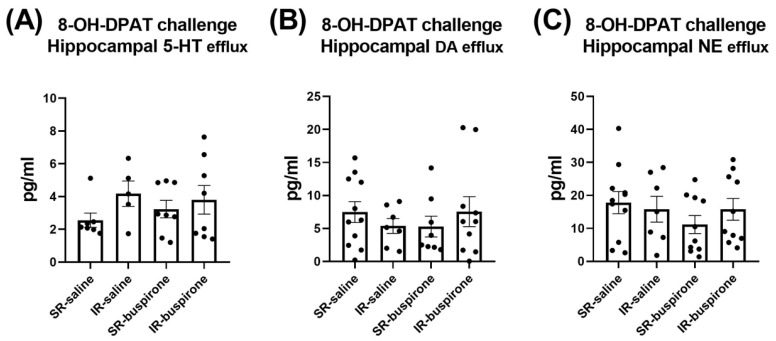
The acute 8-OH-DPAT challenge did not alter the efflux of 5-HT, DA, and NE in the hippocampus. The data are presented as the mean ± SEM. *n* = 5–11 for each group.

For the baseline extracellular levels of 5-HT, DA, and NE among the groups of SR-saline, IR-saline, SR-buspirone, and IR-buspirone, the two-way ANOVA revealed no significant interaction or main effect between the “IR” and “buspirone” (Figure 7A–C). For the extracellular levels of the 5-HT, DA, and the NE acute 8-OH-DAPT challenge, the two-way ANOVA also revealed no significant interaction or main effect between the “IR” and “buspirone” (Figure 8A–C).

**Figure 9:**
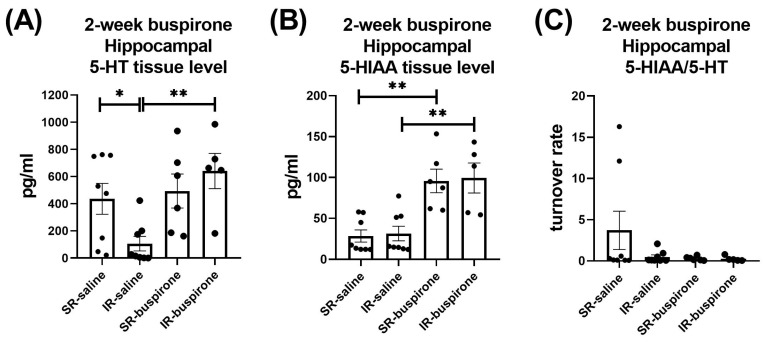
The 2-week buspirone treatment reversed the decrease in hippocampal 5-HT tissue level. It also increased the hippocampal 5-HIAA tissue level in both the SR and IR groups. The data are presented as the mean ± SEM. *n* = 5–8 for each group. * *p* < 0.05, ** *p* < 0.01.

For the hippocampal 5-HT level after the 2-week buspirone treatment, the two-way ANOVA revealed a significant interaction between the “IR” and “buspirone” (F_(1, 23)_ = 5.017, *p* = 0.035). This interaction was driven by the differences observed between the SR-saline and IR-saline groups (F_(1, 23)_ = 5.91, *p* < 0.05), and the IR-saline and IR-buspirone groups (F_(1, 23)_ = 11.948, *p* < 0.01) (Figure 9A). For the hippocampal 5-HIAA level after the 2-week buspirone treatment, the two-way ANOVA revealed a significant main effect on the “buspirone” (F_(1, 23)_ = 33.087, *p* < 0.001). This main effect was driven by the differences observed between the SR-saline and SR-buspirone groups (F_(1, 12)_ = 19.904, *p* < 0.01), and the IR-saline and IR-buspirone groups (F_(1, 11)_ = 13.8790, *p* < 0.01) (Figure 9B). For the hippocampal 5-HT turnover rate after the 2-week buspirone treatment, the two-way ANOVA revealed no significant interaction or main effect between the “IR” and “buspirone” (Figure 9C).

**Figure 10:**
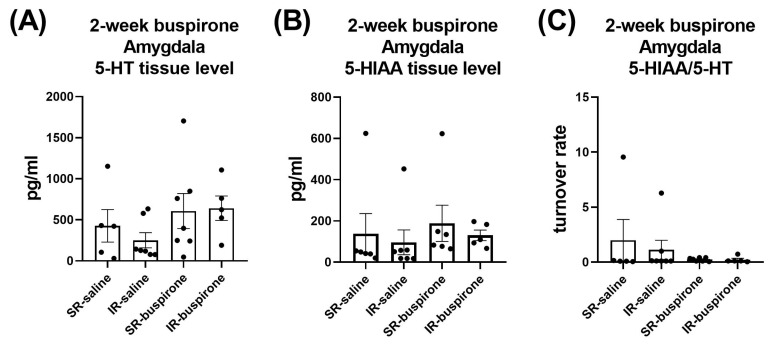
The IR and the 2-week buspirone treatment showed no impact on the tissue levels of 5-HT and 5HIAA or their turnover rate in the amygdala. The data are presented as the mean ± SEM. *n* = 5–8 for each group.

**Figure 11:**
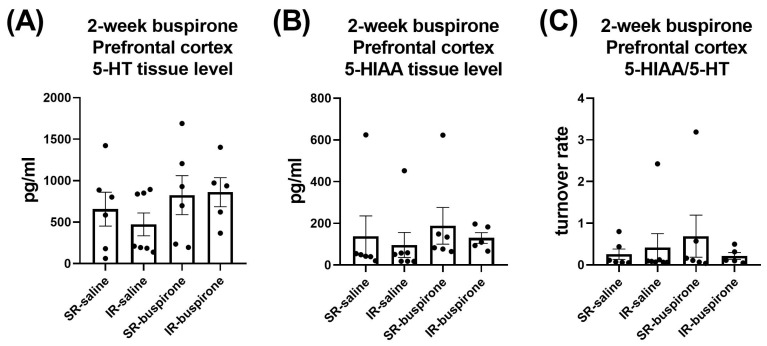
The IR and the 2-week buspirone treatment showed no impact on the tissue levels of 5-HT and 5HIAA or their turnover rate in the prefrontal cortex. The data are presented as the mean ± SEM. *n* = 5–8 for each group.

For the levels of 5-HT, 5-HIAA, and the 5-HT turnover rate in the amygdala and prefrontal cortex after a 2-week buspirone treatment, two-way ANOVA indicated no significant interaction or main effect between “IR” and “buspirone” (Figure 10A–C and Figure 11A–C).

## 3. Discussion

Early life experience serves as a key factor in shaping an individual’s entire life pattern. In a sense, we are all a continuation of our past. Clinical observation reveals that people with a problematic childhood have been found to be at high risk of stress-associated disorders [21,22]. The present study supports this hypothesis that the long-term stress of social isolation since early life plays an important role in the underlying mechanism of depression. We first confirmed the validity of the IR with its nonspecific locomotor hyperactivity and then demonstrated that (i) the IR rats were immobile from the social controls in the FST, which cannot be reversed by the buspirone administration; (ii) the IR-induced immobility in the FST was fixed by 8-OH-DPAT in the buspirone rats; and (iii) the IR-induced reduction in the 5-HT in the hippocampus can be reversed by the buspirone administration. These major findings are now discussed as follows.

In a broad sense, our results support the hypothesis that the central serotonergic system can be a target following long-term social deprivation since early life [23]. Previously, IR rats were reported to exhibit less presynaptic reactivity and greater postsynaptic responsiveness [24,25]. In terms of area-dependent relevance, the present study for a further step demonstrated that the IR rats exhibited lower serotonin concentration in hippocampus, along with the IR-induced impairment of the hippocampal innervation [26]. The reduction in hippocampal 5-HT activity possibly refers to the pathoetiological mechanism of the clinical depressive disorders [27], which is also in line with that of the IR rats that exhibited more immobility in the FST in our results.

The failure of the subchronic buspirone administration to reverse the IR-induced immobility indicates that the partial agonistic effect of the postsynaptic 5-HT receptors per se is less involved in the learned helplessness domain of the depressive mechanism, in contrast to the anhedonia domain (i.e., inability to experience pleasure), which can be corrected by buspirone [28]. It may refer to a symptom-dependent efficacy of the antidepressant treatments [29], also justifying that in the treatment of depression, buspirone appears more eligible to serve as an adjunct with the combination of others to reach better therapeutic efficacy. For example, when combined with selective serotonin reuptake inhibitors (SSRIs), buspirone argumentation facilitates treatment progress by shortening the onset of efficacy, possibly attributable to the desensitization of its presynaptic autoreceptors [30,31]. On the other hand, for the postsynaptic side of the 5-HT projection areas approached in the present study (i.e., hippocampus, amygdala, and prefrontal cortex), the involvement of buspirone lies in its partial agonist effect. Blier and colleagues argued that perhaps by the attribution, postsynaptic 5-HT receptors are able to be selectively activated, which is the key for the antidepressant response [32].

One of the crucial observations in the present study is about the effect of 8-OH-DPAT on the depressive profile. We found that the IR-induced immobility in the FST can be fixed by 8-OH-DPAT in buspirone rats. It was a specific effect, as the immobility-reversing effect only presented in buspirone-pretreated IR rats but not others, indicating the effect of the 8-OH-DPAT to fix the depression-like symptom just presented in conditions where the development-dependent 5-HT incapability (caused by IR) underwent a rather flexible synaptic milieu rendered by the pharmacologically partial agonism of buspirone. This is interesting as it suggests that postsynaptic agonism per se does not adequately ensure the therapeutic efficacy of antidepressants, and a collaboration with presynaptic mediation is thus necessary. Our neurochemical data in this regard may be helpful for interpretation.

In the present study, the IR-induced reduction in 5-HT can be reversed by buspirone administration in the hippocampus, but not in others (i.e., prefrontal cortex and amygdala). This is in line with the mainstream knowledge that agents of azapirones, the chemical category to which buspirone belongs, operate postsynaptically at hippocampal 5-HT1A receptors in a partial agonistic manner [33]. As the turnover rate of hippocampal serotonin (i.e., 5-HIAA/5-HT) did not show any difference among the groups, indicating the buspirone administration affects more in terms of the postsynaptic neuronal events than presynaptic metabolic ones, indicative of the functional link between the neurotransmitter and the metabolite was changed [34,35,36].

The milieu for 5-HT neurotransmission following a long-term partial agonism of the postsynaptic side could be changed in an advantageous manner, as the acute challenge of the full agonist (here, the 8-OH-DPAT) to activate the postsynaptic 5-HT1A receptors more easily and thus to yield the antidepressant effects [37]. The advantageous milieu may be present in two manners. It can be desensitization of the presynaptic 5-HT1A receptors as that is relevant to the clinical efficacy of antidepressants [37,38], or it can be partial agonism-induced upregulation at the postsynaptic side, as it appears in the dopaminergic system. For example, the dopaminergic partial agonist aripiprazole leads to upregulation of postsynaptic dopamine receptors [39]. Both ways may help facilitate the effects of the SSRIs on mood. Note that there were no group differences in our microdialysis experiment. It seems that the neurochemical changes in the synaptic cleft become less sensitive to reflect the long-term effective buspirone effects on the presynaptic side if compared with the changes on the postsynaptic side presented in the tissue-level measurement.

Several concerns or limitations should be addressed for not overly interpreting our results. First, our 14-day subchronic buspirone intervention was carried out at the age of adulthood, not the critical period of early life, raising the concern of a mismatch in the etiology and treatment targeting developmental origin. However, it may be more appropriate/practical to manifest the real-world situation in which people have suffered from an ongoing unpleasant environment since their early lives, but always seek help/treatment later, when they become adults [23]. Second, we did not examine the amount of serotonin transporter (SERT), which may interact with the pharmacological profile of buspirone [18,40,41]. Thus, our interpretation should be cautious if concerning the buspirone-associated presynaptic dynamic change.

In summary, the present study supports the hypothesis that the 5-HT1A receptor-linked early life social experience is involved in the depressive mood of later life. Buspirone with its unique pharmacological 5-HT1A profile of presynaptic full agonism and postsynaptic partial agonism, when combined with 5-HT1A activation, may potentially be useful in the treatment of depression of socially developmental origin.

## 4. Material and Methods

### 4.1. Animals

**Figure 12:**
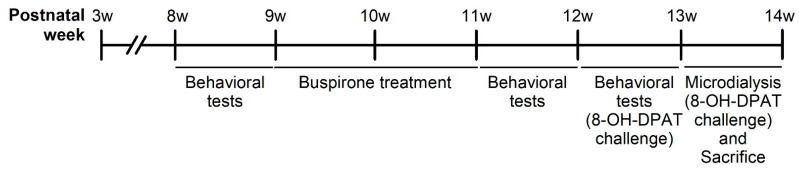
Schematic representation of the experimental design of the present study.

The experimental design is as depicted in Figure 12. A total of 48 male Sprague Dawley (SD) rats (BioLASCO Taiwan Co., Ltd., Taipei, Taiwan) arrived at the National Defense Medical Center (NDMC, Taipei, Taiwan) at postnatal week 3 and were subsequently weaned. The rats were randomly divided into two groups: social rearing (SR, three rats per cage) and IR (one rat per cage), with 24 rats in each group. These conditions were maintained throughout the experiment until they were sacrificed. The first stage of behavioral experiments [i.e., locomotor activity and forced swim test (FST)] was conducted at postnatal week eight to examine the effects of IR. At postnatal week nine, both SR and IR rats were randomly divided into four subgroups: SR-saline, IR-saline, SR-buspirone, and IR-buspirone. They received the corresponding drug regimen for two weeks (postnatal weeks 9–11). The second stage of behavioral experiments was conducted at postnatal week 11 to examine the effects of subchronic buspirone. The third stage of behavioral experiments was conducted at postnatal week 12, as all the rats received the acute 8-OH-DPAT 30 min prior to each behavioral test. During the experimental period, all the rats were housed in a controlled environment with a temperature of 25 °C ± 1 °C, humidity maintained at 50% ± 10%, and a 12 h light-dark cycle (lights on from 07:00 to 19:00). They had access to a standard laboratory chow diet (Ralston Purina, St. Louis, MO, USA) and sterile water ad libitum. The experimental procedures were approved by the NDMC animal care committee (IACUC-20-120, approved on 13 May 2020), with efforts made to minimize the number of animals used and to reduce their suffering during the experiments. All experiments were conducted in accordance with the relevant guidelines and regulations of Taiwan.

### 4.2. IR Procedure

The IR procedure was similar to previous studies [15,42,43]. The IR rats were housed individually, and the SR rats were housed in groups of three rats per cage (the cages were 25 × 45 × 20 cm^3^). The IR rats were placed in a condition where they could smell, see, and hear other rats, but physical contact was prevented. All the rats were minimally handled to avoid unnecessary interruptions.

### 4.3. Drugs

During the postnatal weeks nine to 11, both the SR and IR rats received daily intraperitoneal injections (i.p.) of either 1 mL of buspirone (1 mg/kg) or 1 mL of saline. Additionally, during the third stage of behavioral experiments, all the rats were given acute i.p. administration of 1 mL of 8-OH-DPAT (0.5 mg/kg) 30 min before each behavioral test. All the drugs were freshly prepared before being injected.

### 4.4. Locomotor Activity

The locomotor activity testing apparatus (MED Associates, St. Albans, VT, USA) consisted of a plexiglass chamber (43 × 43 × 30 cm^3^), equipped with 16 photodetectors I/R array and corresponding light sources that emitted photobeams 3 cm apart and 4.5 cm above the chamber floor. During the test, the total travel distance of the rats over 60 min was recorded by a programmed microcomputer (MED Associates, St. Albans, VT, USA).

### 4.5. Forced Swim Test (FST)

For the training stage of the FST, the rats were placed in a plastic cylinder filled with water at 20–25 °C and a depth of 35 cm for 15 min, and they were then removed from the water, dried, and returned to their home cages. The test stage was conducted 24 h later, as the rats were placed back into the water for five minutes under the same conditions, and their behaviors, including immobility, swimming, and climbing, were scored every five seconds (based on which behavior was predominant within each 5 s interval).

### 4.6. Extracellular Levels of 5-HT, DA, and NE in the Hippocampus

The rats were positioned in a stereotaxic apparatus (David Kopf Instruments, Tujunga, CA, USA) using two ear bars after being anesthetized with an intraperitoneal (i.p.) injection of Pentobarbital (1 mg/mL in 0.9% saline, Rhone Merieux, Harlow, UK). A microdialysis probe (MAB, Microbiotech/se AB, Stockholm, Sweden) with a 2 mm active membrane length was implanted into the hippocampus (AP: −5.2 mm, ML: ±5.0 mm, DV: 5.5 mm from the bregma, the midline, and the dura) based on the coordinates described by George P. and Watson C. (2008) [44]. The first round of sampling started one hour after the probe insertion and continued until three dialysate samples were collected, with each sample collected over a period of 30 min. Following this, the second round of three dialysate sampling (30 min for each) was conducted 15 min after 1 mL of 8-OH-DPAT was administered (0.1 mg/kg, i.p.). The artificial cerebrospinal fluid (aCSF), composed of deionized water containing 145 mM NaCl, 1.2 mM CaCl_2_, 2.7 mM KCl, 1 mM MgCl_2_, and 2 mM NaH_2_PO_4_, was perfused through the probe at a rate of 1 μL/min using a syringe pump (CMA-10; CMA Microdialysis, Kista, Sweden). 5-HT, dopamine, and Norepinephrine levels were quantified using an electrochemical detector with a VT-03 cell (Antec, Zoeterwoude, The Netherlands) and a C18 column (3 μm, 100 mm × 2.1 mm, HICHROM, Haryana, India) of high-performance liquid chromatography (HPLC) at a rate of 0.2 mL/min mobile phase (consisted of 100 mM NaH_2_PO_4_, 0.74 mM sodium octane sulfonate, 0.02 mM EDTA, 2 mM KCl, and 10% methanol, adjusted to pH 3.0 using H_3_PO_4_).

### 4.7. Tissue Levels of 5-HT and 5-HIAA in Hippocampus, Amygdala, and Prefrontal Cortex

The rats were euthanized, and their hippocampus, amygdala, and prefrontal cortex were rapidly dissected on an ice-cold surface, according to the coordinates provided by Paxinos and Watson (2008) [44]. The dissected tissues were weighed and homogenized in 0.2 mL of 7 N perchloric acid (Sigma Chemical Industries, Ltd., Saint Louis, MO, USA). The homogenized samples were then centrifuged at 12,000× *g* at 4 °C for 30 min and filtered through a 0.22-μm filter. The concentrations of 5-HT and 5-HIAA were determined using high-performance liquid chromatography (HPLC) with an electrochemical detector (ECD, LC-4C, BAS, West Lafayette, IN, USA). A C^18^, 150 mm × 4.6 mm, 5 μm column (HICHROM, Haryana, India), was utilized, with a flow rate of 1.0 mL/min. The mobile phase consisted of 100 mM NaH_2_PO_4_·H_2_O, 0.74 mM sodium octane sulfonate, 0.02 mM EDTA, 10% methanol, and was adjusted to pH 3.0 using H_3_PO_4_.

### 4.8. Data Analyses

In the present study, both an unpaired t-test and a two-way analysis of variance (ANOVA) were performed. A *p*-value of <0.05 was considered statistically significant. All statistical analyses were performed using the SPSS 16.0 for Windows software (Chicago, IL, USA).

## Data Availability

The datasets used and/or analyzed during the current study are available from the corresponding author on reasonable request.
